# Nivolumab versus placebo as adjuvant therapy for resected stage III melanoma: a propensity weighted indirect treatment comparison and number needed to treat analysis for recurrence-free survival and overall survival

**DOI:** 10.1007/s00262-022-03302-5

**Published:** 2022-10-05

**Authors:** Jeffrey S. Weber, Tayla Poretta, Brian D. Stwalley, Leon A. Sakkal, Ella X. Du, Travis Wang, Yan Chen, Yan Wang, Keith A. Betts, Alexander N. Shoushtari

**Affiliations:** 1 Laura and Isaac Perlmutter Cancer Center at NYU Langone Health, New York, NY USA; 2 Bristol Myers Squibb, Princeton, NJ USA; 3 Analysis Group, Inc., Los Angeles, CA USA; 4 Memorial Sloan Kettering Cancer Center, New York, NY USA; 5 Weill Cornell Medical College, New York, NY USA

**Keywords:** Adjuvant therapy, Nivolumab, Melanoma, Overall survival, Routine surveillance

## Abstract

**Background:**

Recurrence-free survival (RFS) and overall survival (OS) data for adjuvant nivolumab versus placebo (proxy for routine surveillance) in patients with high-risk, resected melanoma are lacking. This post hoc, indirect treatment comparison (ITC) used pooled data from the phase 3 EORTC 18,071 (ipilimumab vs. placebo) and CheckMate 238 (nivolumab vs. ipilimumab) trials to assess RFS and OS with nivolumab versus placebo and the numbers needed to treat (NNT) over 4 years.

**Methods:**

Patients with resected stage IIIB-C cutaneous melanoma (American Joint Committee on Cancer seventh edition) were included. Inverse probability treatment weighting (IPTW) was used to balance baseline characteristics. RFS NNTs were calculated for nivolumab versus ipilimumab and placebo. OS NNTs were calculated for nivolumab versus placebo. To adjust for different post-recurrence treatments, the difference in post-recurrence survival between the two ipilimumab arms was added to OS of the placebo arm.

**Results:**

This ITC included 278, 643, and 365 patients treated with nivolumab, ipilimumab, and placebo, respectively. Following IPTW, nivolumab was associated with improved RFS versus placebo (hazard ratio [HR]: 0.49; 95% confidence interval [CI] 0.39–0.61) and ipilimumab (HR: 0.69; 95% CI 0.56–0.85). RFS NNT was 4.2 for nivolumab versus placebo and 8.9 for nivolumab versus ipilimumab. After post-recurrence survival adjustment, weighted 4-year OS rates were 75.8% for nivolumab and 64.1% for placebo; OS NNT for nivolumab versus placebo was 8.5.

**Conclusions:**

In patients with resected stage IIIB-C cutaneous melanoma in this ITC, nivolumab improved RFS versus placebo and ipilimumab, and OS versus placebo after post-recurrence survival adjustment.

**Supplementary Information:**

The online version contains supplementary material available at 10.1007/s00262-022-03302-5.

## Introduction

Melanoma is the deadliest form of skin cancer [1, 2]. The standard of care for patients with early stage and locally advanced melanoma (i.e., stage I or II disease) involves surgical excision, which can be curative. However, the benefits of surgery are more limited in patients with resectable advanced or metastatic melanoma (stage III or IV disease) [3, 4], leaving a large proportion of this population at high risk of disease recurrence and progression despite surgery [2].

Given that the prognosis among patients with melanoma is worse with advanced disease stage [1], there is a critical need to prevent recurrence. The use of systematic adjuvant treatments following surgery has been shown to decrease the risk of recurrence compared with placebo [3]. In 2015, the cytotoxic T lymphocyte antigen-4 antibody ipilimumab became the first immune checkpoint inhibitor to be approved by the U.S. Food and Drug Administration (FDA) for use as adjuvant therapy among patients with resected stage IIIA-C melanoma (according to the American Joint Committee on Cancer [AJCC] staging system sixth edition) based on results of the phase III EORTC 18,071 trial. In that trial, placebo was used as a proxy for routine surveillance, and by comparison, ipilimumab was associated with significantly improved recurrence-free survival (RFS) and overall survival (OS) [4–6]. However, use of ipilimumab in this population is limited by the risk of immune-related toxicities [4–7]. More recently, the anti-programmed death (PD)-1 antibodies, nivolumab and pembrolizumab, as well as the BRAF/MEK inhibitor combination of dabrafenib plus trametinib (for patients with the *BRAF* V600 mutation), have been shown to significantly improve RFS in the adjuvant setting, with a more manageable toxicity profile than ipilimumab, and are currently considered the standard of care for active interventions [8].

Although the efficacy and safety of ipilimumab, pembrolizumab, and dabrafenib plus trametinib have been compared against placebo as adjuvant melanoma treatment in randomized clinical trials [4–6, 9, 10], no head-to-head trial to date has compared outcomes with nivolumab versus placebo in patients with high-risk resected melanoma. In the pivotal phase III CheckMate 238 trial, nivolumab was compared with ipilimumab for the adjuvant treatment of patients with stage III/IV melanoma who underwent complete resection, whereby it significantly improved RFS at 48 months and had a more favorable toxicity profile at 18 months [11, 12]. Since routine surveillance (i.e., observation) following tumor resection is considered a reasonable option by the National Comprehensive Cancer Network Clinical Practice Guidelines in Oncology (NCCN Guidelines^®^) [13], it is necessary to understand how treatment with nivolumab compares with surveillance in order to inform decision making and improve long-term outcomes.

In 2019, Freeman et al. [14] conducted an indirect treatment comparison (ITC) of nivolumab versus surveillance and ipilimumab using pooled data from EORTC 18,071 and CheckMate 238 [14]. The study estimated the number needed to treat (NNT) and cost per one additional RFS over 24 months and demonstrated that adjuvant nivolumab was both clinically effective and cost-effective compared with surveillance or ipilimumab over 24 months. However, comparative OS data for anti-PD-1 antibody therapy versus placebo are lacking. Given that 4-year data and OS results from CheckMate 238 are now available [12], it is possible to investigate the long-term efficacy of nivolumab versus placebo in an ITC.

The current study aimed to assess the RFS of nivolumab versus placebo and ipilimumab and the OS of nivolumab versus placebo over 4 years, as well as the corresponding NNT in patients with resected stage IIIB-C melanoma using pooled data from EORTC 18,071 and CheckMate 238. The analysis of OS was conducted accounting for differences in post-recurrence treatments between the two trials and its impact on OS. Results from this study are of value to patients, clinicians, payers, and other healthcare decision-makers as they could be used to evaluate the long-term clinical value of adjuvant treatment with nivolumab following surgical resection compared with other options, including routine surveillance.

## Methods

### Data source

Individual patient data were pooled from EORTC 18,071 (NCT00636168) and CheckMate 238 (NCT02388906), both of which were multicenter, double-blind, randomized, phase 3 trials that evaluated RFS as the primary endpoint and OS as a secondary endpoint in patients with high-risk resected melanoma [4, 5, 11, 12].

In EORTC 18,071, patients aged ≥ 18 years with a resected IIIA-C melanoma (based on the AJCC staging system sixth edition, which is identical to AJCC seventh edition for stage III melanoma) were randomly assigned to receive intravenous (IV) ipilimumab (10 mg/kg) or placebo every 3 weeks for 4 doses, then every 12 weeks for up to 3 years [4, 5, 15]. The median follow-up was 5.3 years for the ipilimumab arm and 5.4 years for the placebo arm [5].

In CheckMate 238, patients aged ≥ 15 years with a resected stage IIIB-C or stage IV melanoma (based on the AJCC staging system seventh edition) were randomly assigned to receive IV nivolumab (3 mg/kg) every 2 weeks or IV ipilimumab (10 mg/kg) for 4 doses and then every 12 weeks for up to 1 year [11, 12, 16]. Patients were followed for a median of 4.3 years in the nivolumab arm and 4.2 years in the ipilimumab arm [12].

All study participants provided informed consent and both trials were approved by their respective Institutional Review Boards and were conducted under the Declaration of Helsinki.

### Study population

To perform an ITC, patients with stage IIIB-C cutaneous melanoma who were enrolled in EORTC 18,071 and CheckMate 238 were included in this analysis, as they were the common population of the two trials (EORTC 18,071 and CheckMate 238 excluded patients with stage IV and stage IIIA disease, respectively). Inverse probability treatment weighting (IPTW) was used to balance key baseline characteristics between the participants in both trials. In order to generate a weight for each patient, age, sex, Eastern Cooperative Oncology Group performance status (ECOG PS), time from surgical resection to randomization, disease stage at baseline, tumor ulceration status, lymph node involvement (macroscopic/microscopic), and baseline lactate dehydrogenase (above or below the upper limit of normal [ULN]) were included in a logistic regression model, with enrollment in CheckMate 238 as the dependent variable. Stabilized weights, which preserve the original sample size in the weighted population [17], were generated and used in this study. After adjustment, baseline characteristics were compared before and after weighting using analysis of variance for continuous variables, and chi-squared tests and Fisher’s exact tests for categorical variables.

### Study outcomes

In this study, RFS was separately compared between nivolumab versus placebo and nivolumab versus ipilimumab, and OS was compared between nivolumab versus placebo. The OS comparison between nivolumab versus ipilimumab was not included in the pooled population due to the differences in post-recurrence treatments available in the EORTC 18,071 and Checkmate 238 trials. RFS was defined as the time between the date of randomization and the date of first recurrence (local, regional, or distant metastasis), new primary melanoma (only in CheckMate 238), or death from any cause, whichever occurred first [5, 12]. Patients without recurrence or death were censored at the date of last evaluable disease assessment prior to (or on the same date of) initiation of subsequent therapy, or on the same date of diagnosis of second nonmelanoma primary cancer (only in CheckMate 238) [5, 12]. OS was defined as the time between date of randomization and the date of death from any cause. Patients without a documentation of death were censored on the last date known to be alive [5, 12].

### RFS rate and NNT per additional recurrence-free survivor

Using the pooled patient data, the weighted RFS curves for nivolumab, placebo, and ipilimumab, were estimated using the Nelson-Aalen estimator [18, 19]. The RFS rates for nivolumab, placebo, and ipilimumab, separately, were estimated at 12, 24, 36, and 48 months. In addition, the hazard ratios (HRs) of recurrence or death comparing nivolumab versus placebo and nivolumab versus ipilimumab, separately, were estimated using a Cox proportional hazards model [20].

NNT represents the number of patients who needed to be treated with a treatment compared with another treatment to obtain one additional beneficial outcome [21]. In this study, NNT per RFS comparing nivolumab versus placebo and nivolumab versus ipilimumab were calculated separately as the reciprocal of the absolute risk reduction in RFS between the intervention and its comparator. The corresponding 95% confidence intervals (CIs) were calculated using the approach described by Altman and Andersen [21]. By definition, a 95% CI including infinity indicates that its corresponding NNT is not statistically significant. To ease the interpretation of the NNT results in this case, negative values in 95% CIs of NNT were converted to number needed to harm (NNTH), and positive values were converted to number needed to benefit (NNTB) [21].

### OS rate and NNT per additional overall survivor

Due to the rapidly evolving treatment landscape, patients included in the two trials likely had access to different therapeutic options post-recurrence. To account for higher post-recurrence survival due to improved subsequent treatments for advanced melanoma in CheckMate 238 compared with EORTC 18,071, the analysis of OS was conducted with adjustment of the post-recurrence survival in the placebo arm of EORTC 18,071 under a partitioned-survival framework. The adjusted post-recurrence survival in the placebo arm of EORTC 18,071 represented the potential post-recurrence survival if the post-recurrence treatment options in the CheckMate 238 had been available during the conduct of EORTC 18,071 (online supplemental Fig. 1). Specifically, the weighted RFS and OS were first estimated for the ipilimumab arms of the two trials using a parametric piecewise exponential model with 4-month intervals. Second, the post-recurrence survival was calculated at each time point for the ipilimumab arms in the two trials by subtracting the weighted RFS from the corresponding OS. Third, the difference in post-recurrence survival between the two ipilimumab arms was added to the observed OS of the placebo arm in the EORTC 18,071 to generate the adjusted OS of the placebo arm. This approach assumed that the difference in post-recurrence survival between the CheckMate 238 and EORTC 18,071 was attributable to differences in the availability of subsequent therapies during the conduct of respective trials. Subsequently, using the adjusted OS of placebo, the adjusted OS rates, as well as the corresponding NNT of nivolumab treatment versus placebo were calculated at 12, 24, 36, and 48 months. NNT was calculated as the reciprocal of the absolute risk reduction in OS and represented the number of patients needed to be treated with the intervention versus its comparator to obtain one additional overall survivor. The corresponding 95% CIs were calculated using a bootstrap approach with 1000 iterations [22].

**Fig. 1:**
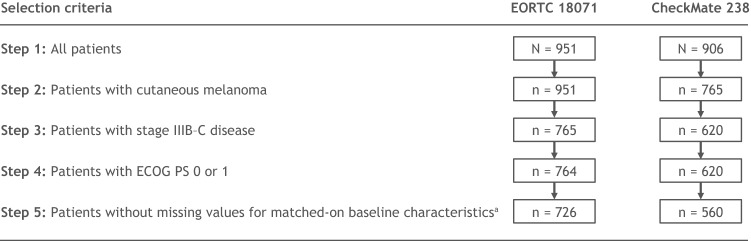
Sample selection prior to pooling EORTC 18,071 and CheckMate 238 data. Thirty-eight patients with missing tumor ulceration status were excluded from EORTC 18,071; 60 patients were excluded from CheckMate 238 due to missing values (15 had missing tumor ulceration status; 36 had missing lymph node involvement status; 7 had missing baseline LDH status; 1 patient had missing tumor ulceration and lymph node involvement status; and 1 patient had missing lymph node involvement and baseline LDH status). *ECOG PS* Eastern Cooperative Oncology Group performance status, *LDH* lactate dehydrogenase

## Results

### Study population and patient characteristics

A total of 726 and 560 patients with stage IIIB-C cutaneous melanoma from EORTC 18,071 and CheckMate 238, respectively, were pooled together, resulting in a final sample size of 1286 patients. Among them, 278 received nivolumab, 643 received ipilimumab, and 365 received placebo (Fig. 1 and Table 1). Key baseline characteristics were well balanced between patients in both trials after IPTW. Mean age was 52 years, 39% were female, and 93% had an ECOG PS of 0. Mean time from surgical resection to randomization was 9.2 weeks.

**Table 1:** Baseline characteristics of patients with stage IIIB-C melanoma in the EORTC 18,071 and CheckMate 238 trials before and after IPTW

	Before weighting			After weighting		
	EORTC 18,071 (*n* = 726)	CheckMate 238 (*n* = 560)	*P* value	EORTC 18,071 (*n* = 726)	CheckMate 238 (*n* = 560)	*P* value
Mean age ± SD, years	51.9 ± 12.9	53.6 ± 13.6	< 0.05^a^	52.4 ± 12.8	52.3 ± 13.8	0.94
Sex, %			< 0.05^a^			0.92
Female	36.1	42.1		39.2	39.5	
Male	63.9	57.9		60.8	60.5	
Race, %			< 0.001^a^			< 0.01^a^
White	99.7	95.7		99.7	95.4	
Other^b^	0.3	4.3		0.3	4.6	
ECOG PS, %			< 0.05^a^			0.71
0	93.9	90.7		93.1	92.6	
1	6.1	9.3		6.9	7.4	
Mean time from resection to randomization ± SD, weeks	9.2 ± 2.2	9.1 ± 2.8	0.18	9.2 ± 2.3	9.2 ± 2.7	0.83
Disease stage at baseline, %			< 0.01^a^			0.62
IIIB	54.3	45.0		50.1	51.6	
IIIC	45.7	55.0		49.9	48.4	
Tumor ulceration, %			< 0.001^a^			0.67
Absent	44.9	59.1		50.9	49.6	
Present	55.1	40.9		49.1	50.4	
Lymph node involvement, %			< 0.01^a^			0.73
Macroscopic	70.5	61.8		66.9	67.9	
Microscopic	29.5	38.2		33.1	32.1	
Baseline LDH, %			< 0.01^a^			0.89
≤ ULN	96.6	93.0		95.2	95.1	
> ULN	3.4	7.0		4.8	4.9	

Baseline characteristics were compared using the chi-square test for categorical variables and ANOVA for continuous variables before weighting. Baseline characteristics after weighting were compared using weighted chi-square test for categorical variables and weighted ANOVA for continuous variables. In the unweighted analysis, the *P* value for race was calculated using Fisher's exact test. In the weighted analysis, the *P* value of race was calculated using a weighted chi-square test

*ANOVA* analysis of variance, *ECOG PS* Eastern Cooperative Oncology Group performance status, *IPTW* inverse probability treatment weighting, *LDH* lactate dehydrogenase, *SD* standard deviation, *ULN* upper limit of normal

^a^Statistically significant at the 95% level

^b^Other race included Asian (1 in EORTC 18,071; 23 in CheckMate 238) and Native Hawaiian or other Pacific Islander (1 each in EORTC 18,071 and CheckMate 238). One patient from the EORTC 18,071 had a missing race entry. Race was not included in the weighting to avoid extreme large weights, since there were only two patients with other races in EORTC 18,071

### RFS for nivolumab versus placebo and nivolumab versus ipilimumab

**Fig. 2:**
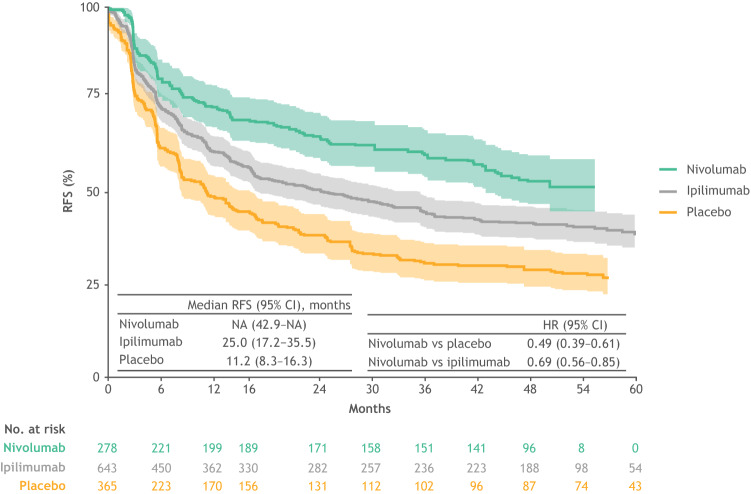
Weighted RFS in the pooled study population by treatment arm. Shaded areas indicate 95% CIs. *CI* confidence interval, *HR* hazard ratio, *RFS* recurrence-free survival

**Fig. 3:**
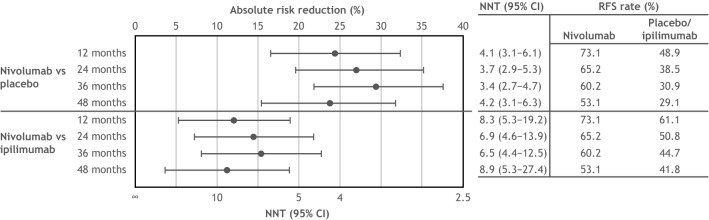
NNT with adjuvant nivolumab versus placebo and nivolumab versus ipilimumab to obtain one additional recurrence-free survivor at 12, 24, 36, and 48 months among patients with stage IIIB-C melanoma. NNT was calculated as the reciprocal of the absolute risk reduction in RFS and represented the number of patients needed to be treated with the intervention versus its comparator to obtain one additional recurrence-free survivor. *CI* confidence interval, *NNT* number needed to treat, *RFS* recurrence-free survival

In the pooled population, patients in the nivolumab arm showed an improvement in RFS compared with those in the placebo arm (HR for recurrence or death: 0.49; 95% CI 0.39–0.61) and ipilimumab arm (HR for recurrence or death: 0.69; 95% CI 0.56–0.85; Fig. 2). Median RFS was not reached in the nivolumab arm and was 25.0 months (95% CI 17.2–35.5) and 11.2 months (95% CI 8.3–16.3) in the ipilimumab and placebo arms, respectively. RFS rates at 48 months in the nivolumab, ipilimumab, and placebo arms were 53.1%, 41.8%, and 29.1%, respectively (Figs. 2 and 3).

At 12 months, the NNT for one additional recurrence-free survivor was 4.1 (95% CI 3.1–6.1) for nivolumab versus placebo, and 8.3 (95% CI 5.3–19.2) for nivolumab versus ipilimumab (Fig. 3). The NNT for nivolumab versus placebo decreased to 3.4 (95% CI 2.7–4.7) at 36 months and subsequently increased to 4.2 (95% CI 3.1–6.3) at 48 months. Similarly, the NNT for nivolumab versus ipilimumab decreased to 6.5 (95% CI 4.4–12.5) at 36 months and subsequently increased to 8.9 (95% CI 5.3–27.4) at 48 months (Fig. 3).

### OS for nivolumab versus placebo

**Fig. 4:**
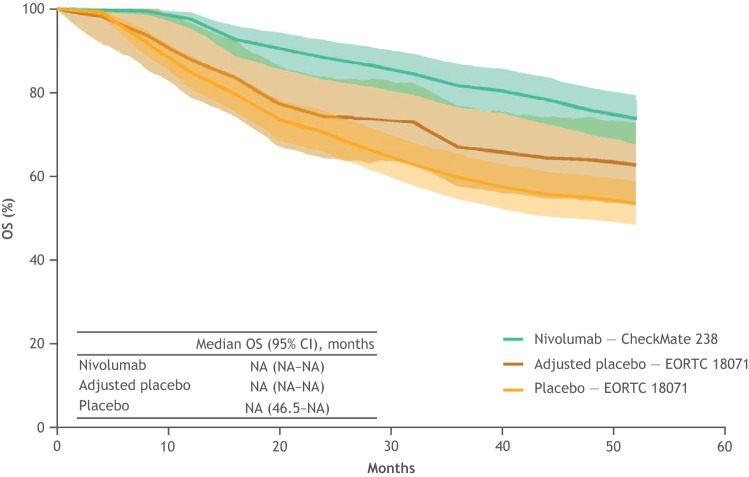
OS for nivolumab and placebo after adjusting for differences in subsequent treatments between the CheckMate 238 and EORTC 18,071 trials. Shaded areas indicate 95% CIs. *CI* confidence interval, *IPTW* inverse probability treatment weighting, *OS* overall survival

**Fig. 5:**
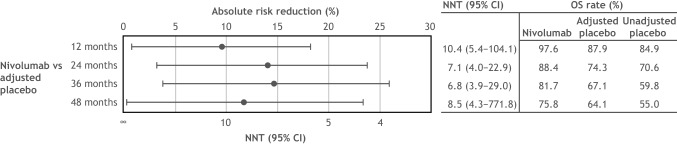
NNT with adjuvant nivolumab versus placebo to obtain one additional overall survivor at 12, 24, 36, and 48 months using pooled data after adjusting for differences in subsequent treatments between the 2 trials. NNT was calculated as the reciprocal of the absolute risk reduction in OS and represented the number of patients needed to be treated with the intervention versus its comparator to obtain one additional overall survivor. *CI* confidence interval, *NNT* number needed to treat, *OS* overall survival

The estimated post-recurrence survival was higher in CheckMate 238 than in EORTC 18,071 by 3.0% at 12 months, 3.7% by 24 months, 7.3% by 36 months, and 9.1% at 48 months. OS outcomes for nivolumab, placebo, and the placebo arm after adjustment are displayed in Fig. 4. Patients in the nivolumab arm showed an improvement in OS compared with those in the placebo arm after adjusting for post-recurrence survival. OS rates for nivolumab were 97.6% at 12 months and 75.8% at 48 months (Fig. 5). Before adjustment, the OS rate for the placebo arm was 84.9% at 12 months and 55.0% at 48 months. After adjusting for post-recurrence survival, the OS rate for the placebo arm was 87.9% at 12 months and 64.1% at 48 months. Median OS was not reached in the nivolumab and the placebo arms, either before or after adjustment.

After adjusting for the post-recurrence survival, the NNT with nivolumab to obtain one additional overall survivor compared with placebo was 10.4 (95% CI 5.4–104.1) at 12 months and decreased to 8.5 (95% CI 4.3–771.8) at 48 months (Fig. 5).

## Discussion

This post hoc ITC, which used pooled, long-term follow-up data from the phase 3 EORTC 18,071 and CheckMate 238 trials [4, 5, 11, 12], suggests that treatment with nivolumab would improve RFS and OS compared with placebo (the proxy for routine surveillance) over 48 months in patients with resected stage IIIB-C (AJCC seventh edition) cutaneous melanoma. The results from this study provide further evidence of the long-term clinical value, in terms of OS and RFS, of adjuvant nivolumab treatment for resected melanoma compared with routine surveillance.

Despite emerging evidence for the benefits of novel adjuvant melanoma treatments, the historical standard approach of surveillance alone may be an option in certain clinical situations and used in real-world practice [23]. According to the NCCN Guidelines^®^, the choice between adjuvant systemic treatment or surveillance alone for stage III sentinel node–positive disease should take into account the patient's risk for disease recurrence and treatment toxicity, and in patients with very-low-risk stage IIIA melanoma (non-ulcerated primary tumor ≤ 2 mm thick, sentinel lymph node metastasis < 1 mm), the benefits of adjuvant therapy may be outweighed by the risk for treatment toxicity [13]. The current study demonstrates that treatment with nivolumab in the adjuvant setting for patients with a high risk for melanoma recurrence (i.e., those with stage IIIB-C resected melanoma) is more efficacious compared with surveillance alone in terms of preventing recurrence or death and extending OS, and that the benefit was maintained or became more prominent over 4 years. Similar clinical benefits with nivolumab over placebo were observed in previous ITC studies including Hemstock et al*.* (HR for recurrence or death: 0.53 [95% CI 0.41–0.68]) [24] and Weber et al*.* (HR for recurrence or death: 0.53 [95% CI 0.42–0.68]; HR for death: 0.63 [95% CI 0.45–0.89]) [25]. Although these studies also utilized EORTC 18,071 and CheckMate 238 data, the HRs are not directly comparable between the studies because of different study populations, data cuts, and statistical methods. However, in contrast to those studies, the current study used a different and potentially more precise ITC methodology, reweighting individual patient data to match baseline characteristics across the two clinical trials. Further, the current study included an NNT analysis. Consequently, this ITC provides additional data that may be important for optimal decision-making among healthcare providers who treat patients with resected melanoma. Nevertheless, the evidence is consistent, and the results of the current study serve to emphasize further the efficacy of long-term nivolumab use in the adjuvant setting for melanoma treatment.

Although this study suggests that adjuvant nivolumab has an OS benefit compared with placebo in high-risk stage IIIB-C cutaneous melanoma, additional studies are needed that evaluate OS with adjuvant nivolumab or other anti-PD-1 antibodies versus placebo in other melanoma subpopulations. Currently, two ongoing trials, CheckMate 76 K (NCT04099251) and KEYNOTE-716 (NCT03553836), are evaluating RFS and OS with adjuvant nivolumab or pembrolizumab versus placebo in patients with stage IIB-C melanoma [26–28]. Interim results from the KEYNOTE-716 trial demonstrated that adjuvant pembrolizumab for patients with resected stage IIB-C melanoma decreased the risk of disease recurrence or death by 39% compared with placebo [29]. Other subpopulations that warrant further research include patients with stage IV melanoma or in-transit metastasis and patients with high-risk mucosal melanoma. Although patients with stage IV disease, in-transit metastasis, and mucosal melanoma were included in CheckMate 238, these patients were not enrolled in EORTC 18,071 and were therefore not included in this analysis. In the ongoing IMMUNED trial, adjuvant therapy with nivolumab plus ipilimumab and adjuvant therapy with nivolumab monotherapy significantly increased RFS compared with placebo in patients with stage IV melanoma with no evidence of disease, although OS data are pending [30].

In addition to survival outcomes, NNT is an intuitive and simple metric used to summarize the investment of time, energy, and resources that clinicians and patients must make to achieve a specific therapeutic goal [31, 32]. Although there is no universally accepted cutoff value to define a desirable NNT, an NNT < 10, which indicates a ≥ 10% absolute risk reduction between active intervention and the reference treatment, is considered the indication of a beneficial response [33, 34]. Of note, treatment-specific NNT estimates should be based on the same probability of response for the reference treatment compared with the interventions [34], and this requirement has been met by the IPTW for the ITC described here. In this study, only an estimated 4 patients needed to be treated with nivolumab instead of surveillance alone to avoid one additional recurrence or death. After adjusting for post-recurrence therapy, it is estimated that 8.5 patients need to be treated by nivolumab to save one life versus placebo at 4 years.

In addition to nivolumab, pembrolizumab (another anti-PD-1 antibody) and the combination of dabrafenib plus trametinib (for patients with *BRAF* V600-activating mutation) have also been approved by the FDA and recommended as adjuvant treatment for resected stage III melanoma, although their clinical trials have different populations. In 2021, Eggermont et al. [35] reported the results of the phase 3 KEYNOTE-054 trial involving patients with resected, high-risk stage III melanoma who were randomly assigned to receive pembrolizumab or placebo. The trial found that the 36-month RFS rate was 63.7% in the pembrolizumab group and 43.5% in the placebo group, which would be equivalent to an NNT of 5.0. In 2020, Dummer et al. [36] reported the results of the phase III COMBI-AD trial, in which patients who had resected stage III melanoma with *BRAF* V600E or V600K mutations were randomly assigned to receive dabrafenib plus trametinib or placebo. At 48 months, the RFS rate in that study was 55% among patients receiving the active treatments and 38% among those receiving placebo, which is equivalent to an NNT of 5.9. In the current study, the NNT for RFS comparing nivolumab with placebo was 3.4 at 36 months and 4.2 at 48 months among patients with stage IIIB-C cutaneous melanoma. However, it should be noted that NNT results for RFS should not be compared across studies because of differences in the trial populations. In 2017, Long et al. [10] reported interim OS results of the phase 3 COMBI-AD trial. Three-year OS rates in COMBI-AD were 86% among patients receiving dabrafenib plus trametinib and 77% among those receiving placebo, which is equivalent to an NNT of 11.1. In the current study, the NNT for OS comparing nivolumab with placebo was 10.4 at 12 months and 8.5 at 48 months among patients with stage IIIB-C cutaneous melanoma. This ITC, using individual patient data and comparing nivolumab with placebo may add additional valuable evidence for physicians deciding on disease management strategies for patients with melanoma who have undergone resection.

This study had certain limitations, some of which are inherent in clinical trials. First, melanoma staging in the two trials was defined based on different editions of the AJCC staging system (i.e., sixth edition in EORTC 18,071 and seventh edition in CheckMate 238); however, given that the main difference between the two editions is in the definition of stage IIIA disease, which was not assessed in this study, this factor is unlikely to influence the comparison. Second, disease recurrence was assessed by an independent review committee in EORTC 18,071 [5] and by the study investigators in CheckMate 238 [12]. The definition of RFS also differed between the two trials, with RFS events including recurrence and death in EORTC 18,071 and recurrence, death, and the development of new primary melanomas in CheckMate 238. However, since new primary melanomas were infrequent, including them is not expected to impact the overall results. In fact, this difference in the RFS definition did not affect the outcomes of a previous ITC with EORTC 18,071 and CheckMate 238 [25]. Third, although key baseline characteristics were well balanced after IPTW and results were robust after adjusting for post-recurrence survival, it is possible that there were unobserved or unadjustable cross-trial differences that may have impacted the results. For example, genetic mutation status (e.g., *BRAF* V600) was not available in EORTC 18,071 because of the timing of the study. In addition, the maximum treatment period was 3 years in EORTC 18,071 and 1 year in CheckMate 238; however, the influence of this difference may have been limited since the median number of ipilimumab doses was four in both trials. Fourth, the sensitivity analysis assumed that the difference in post-recurrence survival between patients included in CheckMate 238 and EORTC 18,071 was attributable to differences in the availability of subsequent therapies during the conduct of the respective trials (and not due to other factors, such as geographical differences). However, it is not possible to test the validity of this assumption. Another limitation is that distant metastasis-free survival (DMFS) was not a prespecified endpoint in the current study, and thus it was not evaluated as an additional measure of efficacy. Other research has found DMFS to support clinical benefits with nivolumab over placebo [24, 25]. Finally, the clinical trial patients included in this study may not have reflected real-world patients who may receive or are eligible to receive adjuvant therapy, because this analysis only examined patients with stage IIIB-C disease (per AJCC seventh edition) and outcomes for nivolumab versus placebo in patients with resectable stage IIIA-IIID or stage IV disease (per AJCC eighth edition) are unknown.

In conclusion, this post hoc ITC provides comparative RFS and OS data for an anti-PD-1 antibody and routine surveillance in the adjuvant melanoma setting. The analysis used long-term follow-up data from the pivotal EORTC 18,071 and Checkmate 238 trials and showed that treatment with nivolumab improved RFS compared with ipilimumab and routine surveillance in the weighted population of patients with resected stage IIIB-C melanoma. After adjusting for post-recurrence therapy, patients treated with nivolumab demonstrated improvements in OS compared with placebo. During a 4-year follow-up period, approximately 8.9 patients needed to be treated with nivolumab compared with ipilimumab in order to obtain one additional recurrence-free survivor, approximately 4.2 patients needed to be treated with nivolumab compared with placebo (i.e., no treatment) to obtain one additional recurrence-free survivor, and 8.5 patients needed to be treated to obtain one additional overall survivor after adjusting for the post-recurrence survival difference. Longer follow-up data from other placebo-controlled adjuvant trials are needed to confirm these findings. With the advent of multiple adjuvant treatment options, the results of this study provide important data for optimal treatment decision-making among healthcare providers who treat patients with resected melanoma.

### Supplementary Information

Below is the link to the electronic supplementary material.

Supplementary file1 (DOCX 188 KB)

## Data Availability

The Bristol Myers Squibb policy on data sharing may be found at https://www.bms.com/researchers-and-partners/independent-research/data-sharing-request-process.html.

## Abbreviations

AJCC: American Joint Committee on Cancer
ANOVA: Analysis of variance
CI: Confidence interval
DMFS: Distant metastasis-free survival
ECOG PS: Eastern Cooperative Oncology Group performance status
FDA: Food and Drug Administration
HR: Hazard ratio
IPTW: Inverse probability treatment weighting
ITC: Indirect treatment comparison
IV: Intravenous
LDH: Lactate dehydrogenase
NCCN: National Comprehensive Cancer Network
NMA: Network meta-analysis
NNT: Number needed to treat
NNTB: Number needed to benefit
NNTH: Number needed to harm
OS: Overall survival
PD: Programmed death
RFS: Recurrence-free survival
SD: Standard deviation
ULN: Upper limit of normal
