# Correction to: Nivolumab versus placebo as adjuvant therapy for resected stage III melanoma: a propensity weighted indirect treatment comparison and number needed to treat analysis for recurrence-free survival and overall survival

**DOI:** 10.1007/s00262-022-03351-w

**Published:** 2022-12-20

**Authors:** Jeffrey S. Weber, Tayla Poretta, Brian D. Stwalley, Leon A. Sakkal, Ella X. Du, Travis Wang, Yan Chen, Yan Wang, Keith A. Betts, Alexander N. Shoushtari

**Affiliations:** 1 Laura and Isaac Perlmutter Cancer Center at NYU Langone Health, New York, NY USA; 2 Bristol Myers Squibb, Princeton, NJ USA; 3 Analysis Group, Inc., Los Angeles, CA USA; 4 Memorial Sloan Kettering Cancer Center, New York, NY USA; 5 Weill Cornell Medical College, New York, NY USA

**Correction to: Cancer Immunology, Immunotherapy** 10.1007/s00262-022-03302-5

The article Nivolumab versus placebo as adjuvant therapy for resected stage III melanoma: a propensity weighted indirect treatment comparison and number needed to treat analysis for recurrence‑free survival and overall survival, written by Jeffrey S. Weber, Tayla Poretta, Brian D. Stwalley, Leon A. Sakkal, Ella X. Du, Travis Wang, Yan Chen, Yan Wang, Keith A. Betts and Alexander N. Shoushtari, was originally published electronically on the publisher’s internet portal on 05 October 2022 without open access. With the author(s)’ decision to opt for Open Choice the copyright of the article changed on 28 November 2022 to © The Authors 2022 and this article is licensed under a Creative Commons Attribution 4.0 International License, which permits use, sharing, adaptation, distribution and reproduction in any medium or format, as long as you give appropriate credit to the original author(s) and the source, provide a link to the Creative Commons licence, and indicate if changes were made. The images or other third party material in this article are included in the article's Creative Commons licence, unless indicated otherwise in a credit line to the material. If material is not included in the article's Creative Commons licence and your intended use is not permitted by statutory regulation or exceeds the permitted use, you will need to obtain permission directly from the copyright holder. To view a copy of this licence, visit http://creativecommons.org/licenses/by/4.0/.

The original article has been corrected.

