# Supplementary material for: Nivolumab versus placebo as adjuvant therapy for resected stage III melanoma: a propensity weighted indirect treatment comparison and number needed to treat analysis for recurrence-free survival and overall survival
Source: Cancer Immunol Immunother. 2022 Oct 5;72(4):945–54. doi: 10.1007/s00262-022-03302-5 (PMC10025222; doi:10.1007/s00262-022-03302-5)

# Supplement

**Nivolumab versus placebo as adjuvant therapy for resected stage III melanoma: a propensity weighted indirect treatment comparison and number needed to treat analysis for recurrence-free survival and overall survival**

Jeffrey S. Weber,^1^ Tayla Poretta,^2^ Brian D. Stwalley,^2^ Leon A. Sakkal,^2^ Ella X. Du,^3^ Travis Wang,^3^ Yan Chen,^3^ Yan Wang,^3^ Keith A. Betts,^3^ Alexander N. Shoushtari^4, 5^

^1^Laura and Isaac Perlmutter Cancer Center at NYU Langone Health, New York, NY, USA; ^2^Bristol Myers Squibb, Princeton, NJ, USA: ^3^Analysis Group, Inc., Los Angeles, CA, USA; ^4^Memorial Sloan Kettering Cancer Center, New York, NY, USA; ^5^Weill Cornell Medical College, New York, NY, USA

**Supplemental Figure 1.** Analytical steps for comparing OS for nivolumab versus placebo by adjusting for difference in subsequent treatments between the CheckMate 238 and EORTC 18071 trials. OS, overall survival; IPTW, inverse probability treatment weighting; NNT, number needed to treat; RFS, recurrence-free survival.


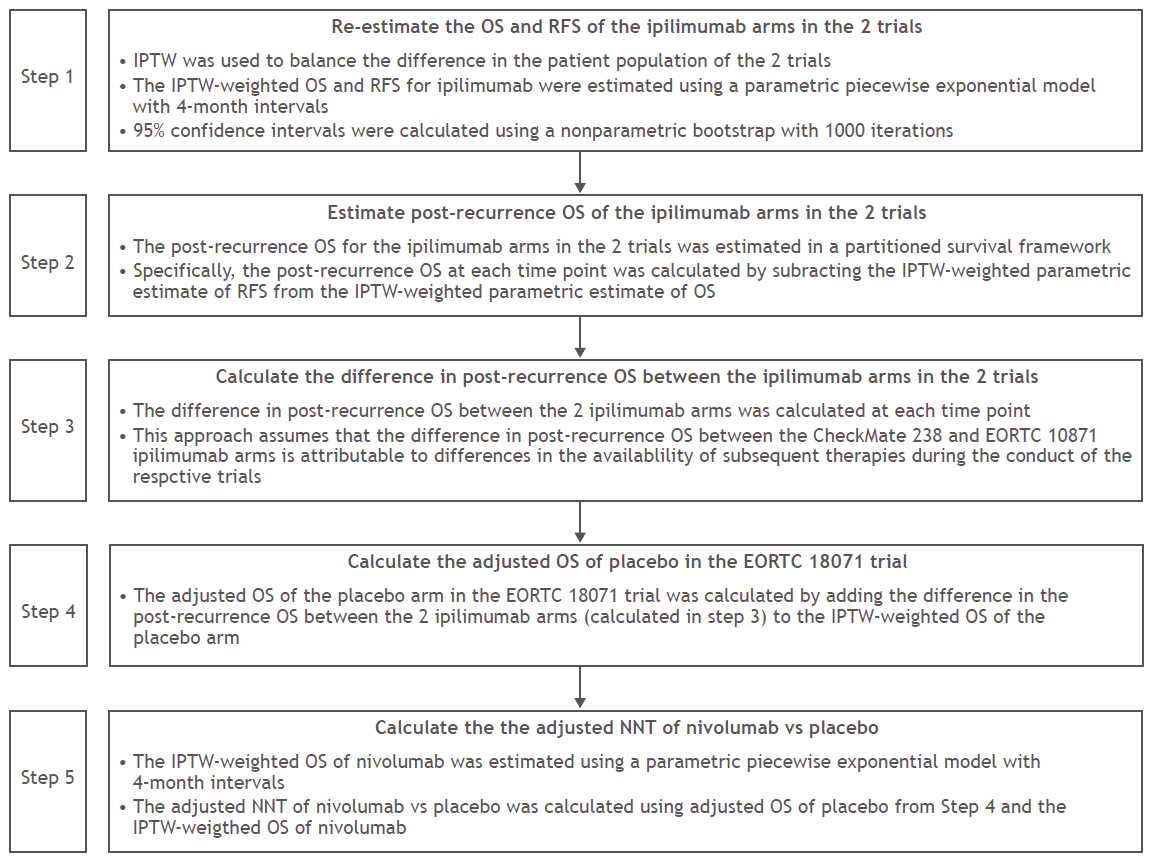

Supplement: Supplementary file 1 — Supplementary file1 (DOCX 188 KB) [file 262_2022_3302_MOESM1_ESM.docx]
